# Discovery of a Low Toxicity O-GlcNAc Transferase (OGT) Inhibitor by Structure-based Virtual Screening of Natural Products

**DOI:** 10.1038/s41598-017-12522-0

**Published:** 2017-09-26

**Authors:** Yubo Liu, Yang Ren, Yu Cao, Huang Huang, Qiong Wu, Wenli Li, Sijin Wu, Jianing Zhang

**Affiliations:** 1 School of Life Science & Medicine, Dalian University of Technology, Panjin, China; 2 School of Life Science & Biotechnology, Dalian University of Technology, Dalian, China

## Abstract

O-GlcNAc transferase (OGT) plays an important role in regulating numerous cellular processes through reversible post-translational modification of nuclear and cytoplasmic proteins. However, the function of O-GlcNAcylation is still not well understood. Cell permeable OGT inhibitors are needed to manipulate O-GlcNAcylation levels and clarify the regulatory mechanism of this modification. Here, we report a specific natural-product OGT inhibitor (**L01**), which was identified from a structure-based virtual screening analysis. **L01** inhibited O-GlcNAcylation both *in vitro* and in cells without significantly altering cell surface glycans. Molecular dynamics and site-directed mutagenesis indicated a new binding mechanism in which **L01** could interact with Asn557 near the UDP binding pocket of OGT. This residue may contribute to the specificity of **L01**. Furthermore, as a specific OGT inhibitor, **L01** produced low toxicity in cellular and zebrafish models. The identification of **L01** validates structure-based virtual screening approaches for the discovery of OGT inhibitors. **L01** can also serve as a chemical tool to further characterize O-GlcNAcylation functions or a new molecular core for structure-activity relationship studies to optimize the biochemical potencies.

## Introduction

O-GlcNAc transferase (OGT) mediates protein O-GlcNAcylation, a ubiquitous posttranslational modification characterized by the attachment of N-acetylglucosamine moieties from uridine diphosphate N-acetylglucosamine (UDP-GlcNAc) to serine or threonine residues of nuclear and cytoplasmic proteins in multicellular eukaryotes^1^. Results from global proteomic experiments have shown that hundreds proteins involved in a wide range of cellular functions, are dynamically and reversibly modified with O-GlcNAc^2,3^. O-GlcNAcylation has been proposed to modulate gene transcription, signal transduction, cellular stress response and protein stability. Altered protein O-GlcNAc profiles have been associated with the occurrence and development of numerous critical diseases, including diabetes, cardiovascular disease, cancer, Alzheimer’s disease and other neurodegenerative disorders^4–6^. Aberrant OGT activity was reported to be a feature of several illnesses including cancer^7^, and selective small-molecule OGT inhibitors would be useful as probes to investigate the primary biological functions of O-GlcNAc and could validate OGT as a therapeutic target. Therefore, OGT inhibitors that demonstrate selective, on-target inhibition and low toxicity in cells are required.

However, most of the reported compounds in the last few years have not been shown to inhibit OGT effectively or selectively^8^. The uracil analogue Alloxan was the first reported OGT inhibitor but was questionable due to cellular toxicity and off-target effects^9^. Ac_4_-5S-GlcNAc and BADGP are mimics of the OGT donor substrate UDP-GlcNAc. These two compounds affected other glycosyltransferases by either direct or indirect inhibition, which induced abnormal cell surface glycan expression^10–12^. Other substrate mimics were also proposed to inhibit OGT *in vitro*, but they were not cell-permeable and therefore were ineffective in cells^13^. More recently, OGT inhibitors discovered by high-throughput screening showed some utility in cells. BZX is a covalent inhibitor identified by this strategy. However, it was shown to be potentially toxic and have off-target effects^14^. Similarly, another cell-permeable inhibitor, OSMI-1, was recently designed and shown to minimally affect cell surface glycan synthesis. Nevertheless, it decreased cell viability by approximately 50% after treatment^10^. Therefore, future work is needed to develop cell-permeable, potent and low toxicity OGT inhibitors with novel scaffolds.

Natural products have long served as potentially rich source of novel bioactive scaffolds that display remarkable chemical diversity in structure and function^15^. An assessment of all FDA-approved new molecular entities reveals that natural products and their derivatives represent over one-third of all new molecular entities^16^. Moreover, modified natural products have shown special selectivity toward disease-related targets with cellular permeability^17^. Nevertheless, no natural-product OGT inhibitor has been reported. Structure-based virtual screening has emerged as an efficient strategy in natural product drug discovery, complementing conventional random screening^18^. By eliminating inactive non-binders in silico, the numbers of compounds to be tested *in vitro* can be dramatically reduced. As the crystal structure of human OGT in complex with the donor sugar substrate UDP-GlcNAc has been used to characterize enzyme-substrate interactions^19^, it is available for virtual screening for the rapid and efficient discovery of lead natural compounds against OGT.

Based on these data, a structure-based high-throughput virtual screening was carried out. The ADME-Tox (absorption, distribution, metabolism, excretion and toxicity) prediction was applied to evaluate the properties of the small molecule candidates before screening, and twelve compounds of the top ranked 200 in silico were preliminarily tested for inhibition of OGT activity. **L01** was further selected to undertake an exploratory study of its *in vitro* OGT inhibitory effects. Moreover, we demonstrated that **L01** specifically inhibited O-GlcNAcylation in cells without significant acute toxicity *in vivo*. This potential lead compound identified from the current study can be useful in the design and development of a novel OGT inhibitor.

## Results

### Identification of a novel OGT inhibitor from a virtual screen

**Figure 1:**
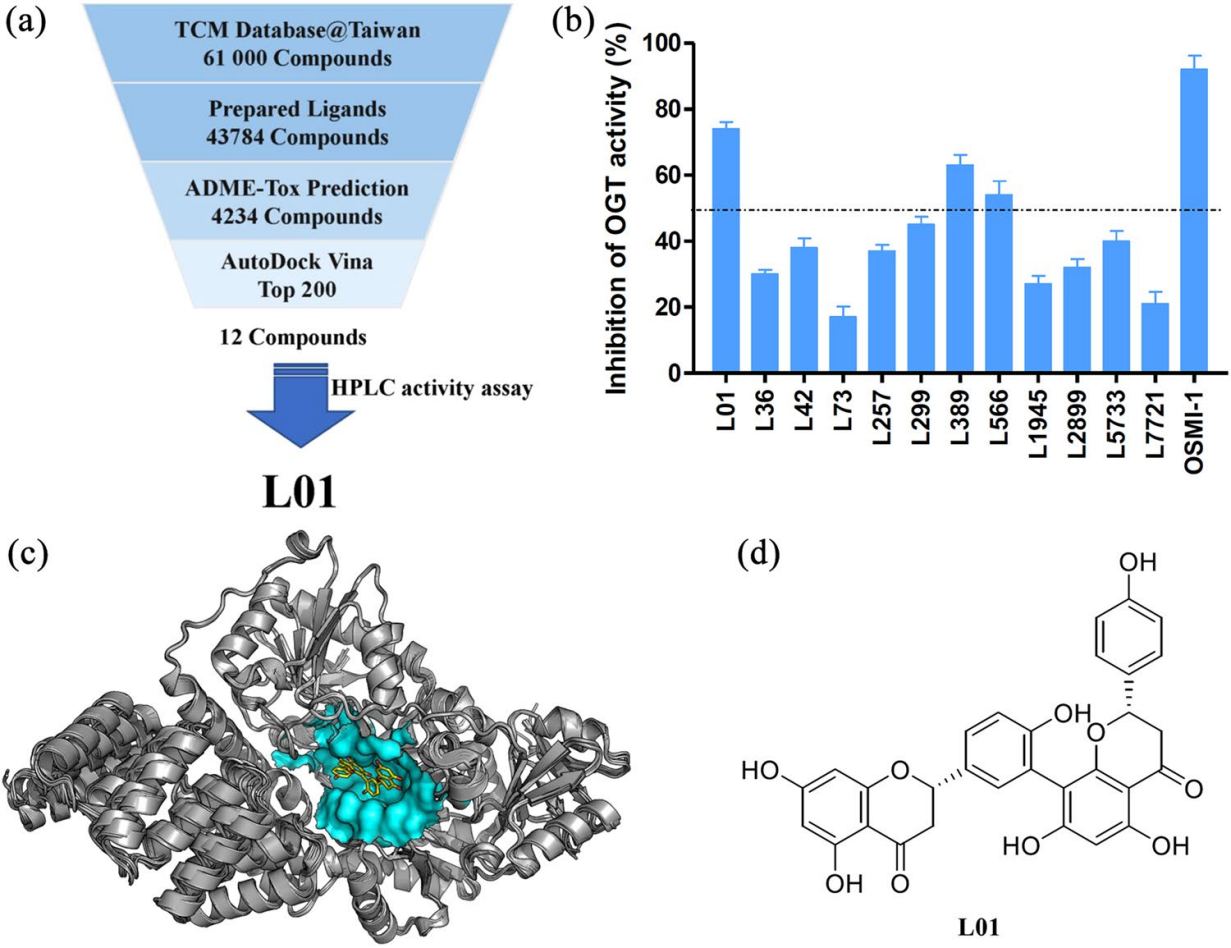
Structural-based virtual screening protocol and structure of **L01**. (**a**) Flowchart for the identification of **L01**. (**b**) Effect of candidates on OGT activities. All the compounds were used at 100 μM. OSMI-1 was used as a positive control. Enzyme activity was tested by using HPLC-based activity assay and normalized to the DMSO control (data represent the mean ± s.e.m., n = 3). (**c**) The human OGT ensemble structures and the binding pose of **L01** with the best score. The residues of binding pocket are shown by surface. (**d**) The chemical structure of **L01**.

To date, although various classes of OGT inhibitors were reported^8^, virtual screening has not been used in OGT inhibitor discovery. In this study, structural-based virtual screening was conducted following the strategy shown in Fig. 1a. Sixteen X-ray structures of the human OGT binding to UDP or UDP-GlcNAc that represent various conformational changes of OGT were used as the receptor library for virtual screening. The search area for the screening was set near the UDP binding pocket of OGT^19^. A total of approximately 61,000 compounds from TCM Database@Taiwan, a chemical library of natural products and natural product-like molecules, were screened. ADME-Tox and other properties were predicted for all molecules by the FAF-Drugs server. Then, 4234 compounds were assessed by the virtual screening with the 16 structures in the OGT structures-ensemble library by Autodock Vina. The top 200 common compounds with high average Vina scores for each OGT structure were selected for further study.

After visual inspection considering the docking poses, structural diversity and novelty, 12 candidates (structures are shown in Supporting Information Table S1) from the 200 compounds were purchased. To evaluate their ability to inhibit OGT activity *in vitro*, these compounds were tested in a preliminary HPLC assay that detects the transfer of GlcNAc to a well-characterized OGT peptide substrate CKII (Fig. 1b). From these results, the biflavonoid derivative **L01** (Fig. 1c and d) emerged as the top candidate.

### **L01** inhibits OGT *in vitro*

**Figure 2:**
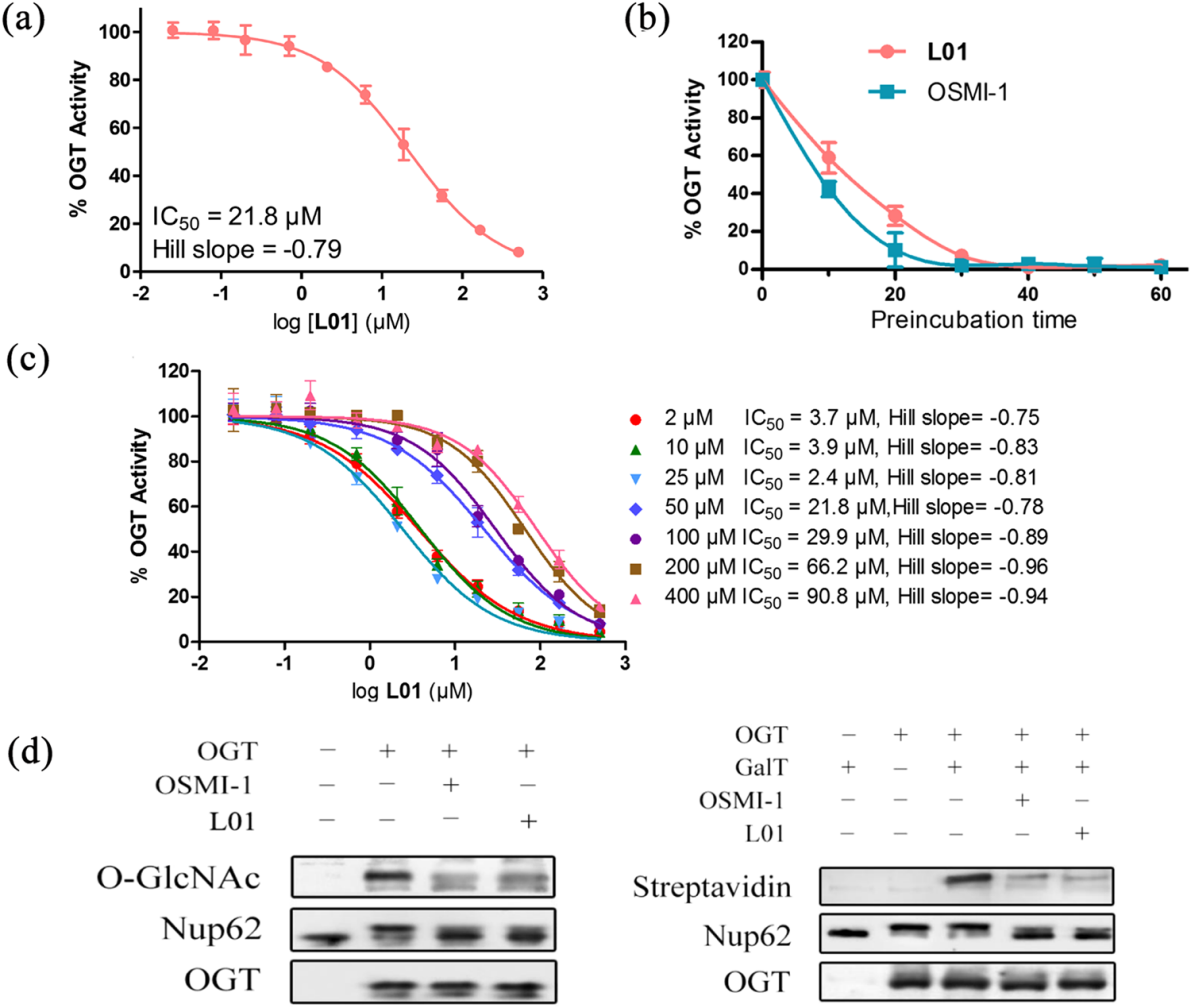
**L01** inhibited OGT in an enzyme-based assay. (**a**) **L01** inhibited OGT activity in a dose-dependent manner. The indicated doses (0.25–500 μM) of **L01** were used in the UDP-Glo assay (data represent the mean ± s.e.m., n = 3). (**b**) **L01** (50 μM) inhibited OGT in a time-dependent manner in the UDP-Glo assay (data represent the mean ± s.e.m., n = 3). OSMI-1 (50 μM) was used as a positive control. (**c**) Inhibition mode of OGT by **L01**. Various UDP-GlcNAc concentrations (2, 10, 25, 50, 100, 200 and 400 μM) were used in the UDP-Glo assay (data represent the mean ± s.e.m., n = 3). (**d**,**e**) **L01** (50 μM) inhibited O-GlcNAcylation of recombinant Nup62 in a cell-free reaction system. Purified Nup62 and OGT were supplied with UDP-GlcNAc, and compounds were incubated at 37 °C for 1 h. OSMI-1 (50 μM) was used as a positive control. (**d**) Western blots were used to detected the O-GlcNAcylation and the shift of Nup62. (**e**) Alternatively, the reaction system was incubated with UDP-GalNAz in the absence (−) or presence (+) of mutant GalT1, and chemoselectively labeled. O-GlcNAcylation of Nup62 was detected by Streptavidin.

To confirm the ability of **L01** to inhibit OGT, we assessed its inhibitory activity using a UDP-Glo glycosyltransferase assay, which measures the UDP production when GlcNAc is transferred from UDP-GlcNAc to a peptide acceptor (CKII)^10^. A dose-response experiment was performed to determine the potency of **L01** in inhibiting the GlcNAc transfer activity of OGT. **L01** effectively inhibited OGT with an IC_50_ of 21.8 μM (Fig. 2a). A similar IC_50_ value (20.2 μM) was obtained using the HPLC assay (Fig. S1a). As a positive control, a previously established OGT inhibitor, OSMI-1, was also tested in parallel. OSMI-1 was recently reported to have a higher specificity against OGT than other extant compounds. In an *in vitro* assay, OSMI-1 had a 20-fold lower IC_50_ value compared to ST045849 (a commercially available OGT inhibitor). OSMI-1 has recently been used to study the role of O-GlcNAc in the replication of herpes simplex virus (HSV), suggesting the effective OGT inhibition of this compound^20^. In our experimental condition, the IC_50_ values of OSMI-1 in the UDP-Glo and HPLC assays were 3.5 and 6.2 μM, respectively (Fig. S1a and b). In addition, **L01** was found to inactivate OGT in a time-dependent manner when we assessed OGT activity after preincubation with **L01** for 30 min before addition of the substrate (Fig. 2b, OSMI-1 was used as a control). This abnormal time-dependent inhibition of OGT indicated that other inhibition mode may exist. The higher IC_50_ and longer working time of **L01** compared with OSMI-1 also suggested that the affinity of **L01** to OGT was weaker than OSMI-1. Additional experiments were performed to exclude potential nonspecific OGT inhibition of **L01**, such as through redox, aggregation, and irreversible inhibition (Fig. S1b and c). Then, we investigated the inhibition mode of **L01**. The shift in IC_50_ values for **L01** did not correlate to low concentrations UDP-GlcNAc (Fig. 2c). The IC_50_ values of **L01** increased in a stepwise fashion with the increasing UDP-GlcNAc concentrations and IC_50_ values correlated to UDP-GlcNAc when UDP-GlcNAc was above 25 μM, indicating that **L01** may not completely act as a UDP mimic. Consistently, under saturating CKII peptide, variable UDP-GlcNAc and initial rate conditions, the V_max_ for O-GlcNAcylation decreased with the increasing concentration of **L01** (Fig. S2a).

We next probed the inhibition of O-GlcNAc levels *in vitro* on a purified protein acceptor nucleoporin 62 (Nup62, *Rattus norvegicus*), which is a heavily glycosylated component of the nuclear pore^21^. Immunoblot analysis using an anti-O-GlcNAc antibody (CTD110.6) showed that O-GlcNAc modification of recombinant Nup62 by purified OGT in a cell free reaction system was decreased along with a shift in molecular weight by **L01** treatment (Fig. 2d, OSMI-1 was used as a control). Similar results were obtained using a commercially available enzymatic method (O-GlcNAc Enzymatic Labeling System) that involves chemoselective ligation of biotin to terminal GlcNAc residues (Fig. 2e). We also performed a parallel experiment with another glycosyltransferase, ppGalNAc-T2, to evaluate the specificity of **L01**. This compound did not strongly inhibit ppGalNAcT2 (IC_50_ > 500 μM, Fig. S3a). Moreover, **L01** did not affect the activity of other cellular enzymes (glucose oxidase, lactic dehydrogenase and thioredoxin reductase, Fig. S3b), suggesting that **L01** cannot interact with these proteins and affect their activity in a non-specific mode. These results suggest that **L01** effectively blocks the glycosylation by OGT *in vitro* and it is therefore a potential OGT inhibitor.

### **L01** putatively interacts with residues in the UDP-binding pocket of OGT

**Figure 3:**
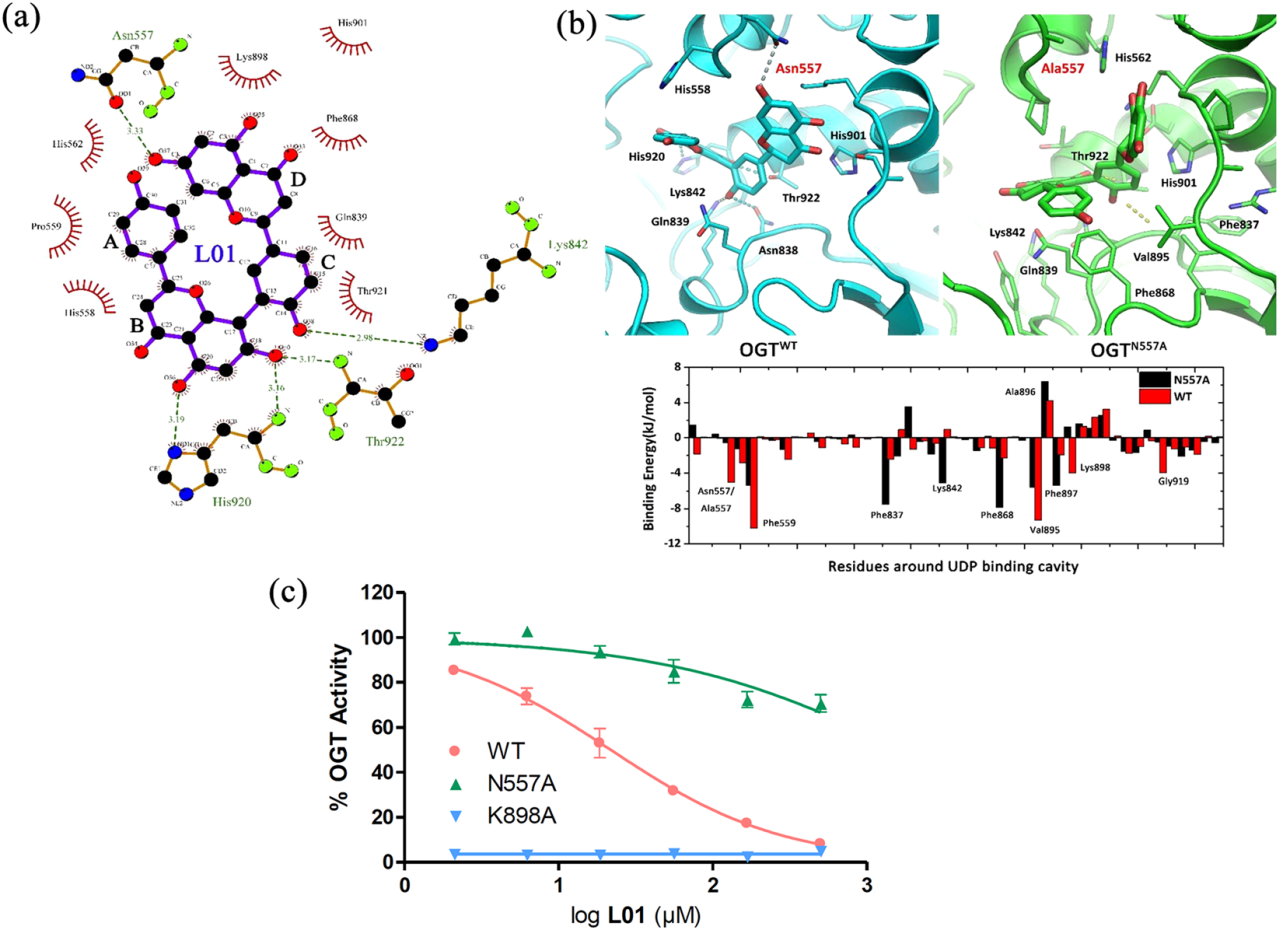
Molecular modeling and mutation analyses. (**a**) Binding mode of **L01-**OGT from the biggest cluster of MD trajectory. (**b**) N557A mutation simulation and per-residue binding energy contribution calculated for the residues of the binding cavity of UDP. H-bonds are shown in dotted lines. (**c**) OGT inhibition curve of **L01** to purified OGT^WT^ and other two mutants (N557A, K898A) by UDP-Glo assay. Indicated doses of **L01** were used (data represent the mean ± s.e.m., n = 3).

To address the structural mechanism of the strong inhibitory activities of the newly identified natural-product inhibitor with OGT, the binding mode of the **L01**-OGT complex was studied by molecular dynamics (MD) simulation and free energy calculation methods (Fig. S4 and Table S2). All MD simulations adopted the OGT structure 3PE3^19^. MD results showed that the binding site of **L01** in OGT was similar to that of UDP (Fig. S5a). The B and C rings of **L01** were deep inside the UDP binding cavity, which mapped onto the phosphate groups of UDP, forming hydrogen bonds with Lys842, His920 and Thr922. The D ring of **L01** was predicted to be located at the entrance of the UDP binding pocket, where the uridine group of UDP was located (Fig. 3a). Compared with the binding free energy of UDP (−554.772 kJ/mol), **L01** (−100.334 kJ/mol, Table S2) did not show full inhibition of OGT activity, indicating that the IC_50_ of **L01** was reasonably high.

Interestingly, another hydrogen bond between Asn557 and hydroxyl groups in the D ring made a strong contribution to the interaction between **L01** and OGT. This bond has not been previously described in the OGT-UDP simulations or crystal structures. Therefore, we estimated the impact of an Asn557 to Ala mutation (N557A) in silico and *in vitro*. As expected, the binding residues of **L01** were substantially different between wild type OGT (OGT^WT^) and N557A OGT (OGT^N557A^) (Fig. 3b). Moreover, this mutation also resulted in a significant decrease in the binding energies of **L01** (OGT^WT^ = −100.334 kJ/mol, OGT^N557A^ = −60.005 kJ/mol) but not UDP (Table S2). Site-directed mutagenesis of this residue followed by an *in vitro* glycosylation assay showed similar results, indicating that **L01** interacts with Asn557 near the UDP-GlcNAc-binding pocket of OGT (Fig. 3c). Additional kinetic assay showed that **L01** was insensitive to UDP-GlcNAc concentration in OGT^N557A^ (Fig. S2b). These dates indicated that N557A mutation inhibited the binding of **L01** to OGT and might affect the competition of this compound with UDP-GlcNAc. Consistent with previous reports^22^, mutation of Lys898 (K898A), which is involved in UDP binding, results in the complete inactivation of the OGT activity.

### **L01** is an efficient, specific OGT inhibitor in cells

**Figure 4:**
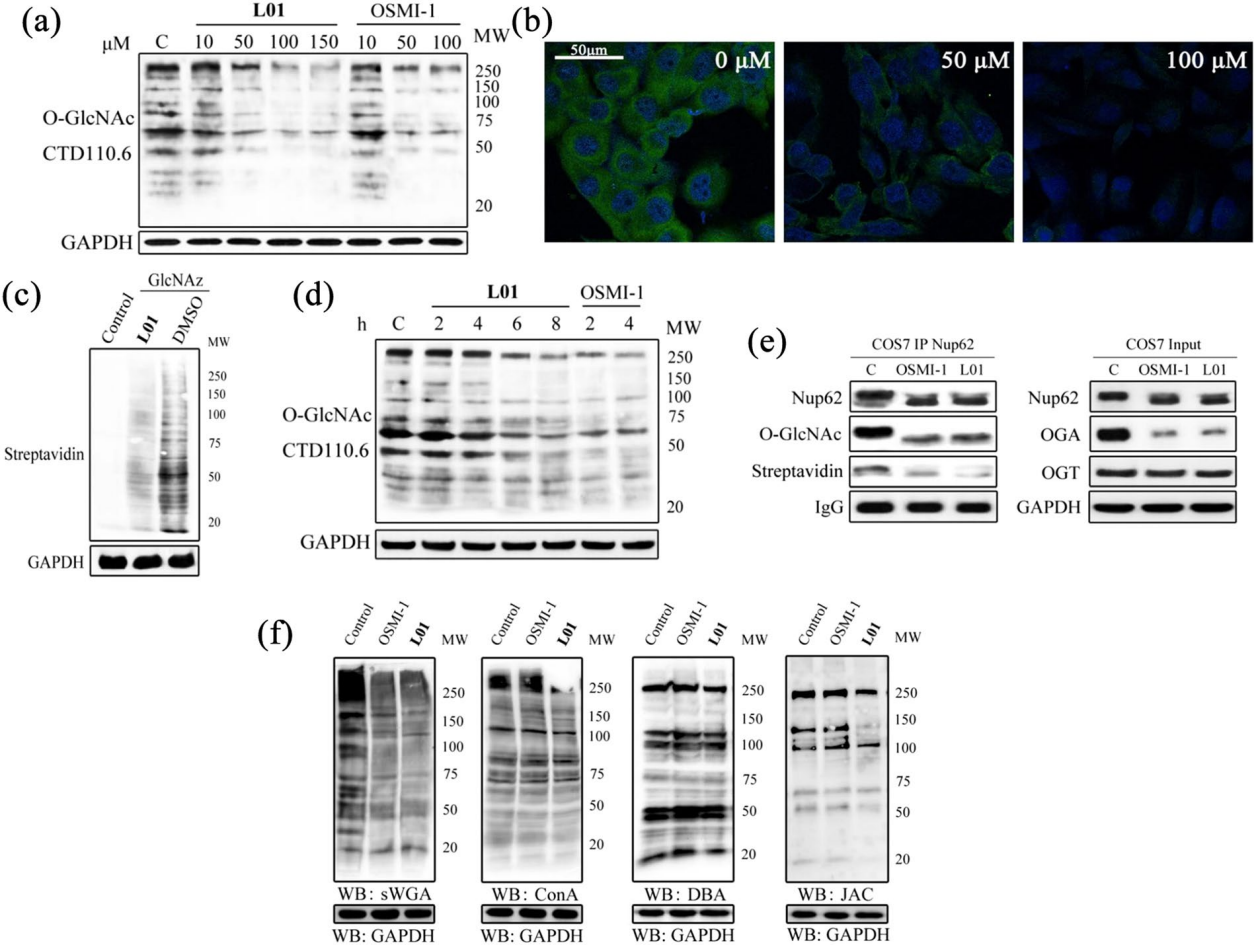
**L01** acts in cells to inhibit OGT and does not grossly perturb cell-surface glycan structures. (**a**) Western blots of COS7 cell lysates after **L01** treatment at different doses (0–150 μM) for 24 h. OSMI-1 (0–100 μM) was used as a positive control. (**b**) Immunocytochemistry of COS7 cells treated with 0, 50 μM or 100 μM **L01** for 24 h. Immunoreactivity from antibody to O-GlcNAc CTD110.6 is green and DAPI is blue. (**c**) COS7 cells were metabolic labeled with UDP-GlcNAz (GlcNAz) and then administered with **L01** (100 μM) or DMSO for 24 h. COS7 cells which did not metabolic label were used as a negative control. All the cells were harvested and then underwent Click reaction with biotin-alkyne. Blots were probed with streptavidin-HRP. (**d**) Western blots of COS7 cell lysates after **L01** treatment at 50 μM for indicated times. OSMI-1 (50 μM) was used as a positive control. (**e**) Western blots of immunoprecipitated Nup62 from cell lysates after DMSO (C) or 100 μM **L01** treatment for 24 h. After immunoprecipitation, Nup62 was incubated with UDP-GalNAz in the presence of mutant GalT, and chemoselectively labeled. (**f**) Lectin blots of COS7 cell lysates after **L01** or OSMI-1 treatment at 50 μM for 24 h.

Given that **L01** could block OGT glycosylation activity in a cell-free system, we subsequently investigated the ability of **L01** to antagonize OGT activity in living cells. After 4 h glucose starvation, COS7 cells were incubated with **L01** ranging from 10 to 150 μM for 6 h. **L01** attenuated global O-GlcNAcylation in a dose-dependent manner as evaluated by immunoblotting using CTD110.6 (Fig. 4a). **L01** was less effective in inhibiting cellular O-GlcNAcylation than OSMI-1 at 10 μM. The maximal effect of **L01** was observed at 100 μM. By contrast, due to the limited aqueous solubility, concentrations higher than 50 μM of OSMI-1 did not decrease O-GlcNAc levels further. Although **L01** showed weaker inhibition of OGT than OSMI-1 *in vitro*, it was comparable with OSMI-1 in reducing of global O-GlcNAc levels in living cells at 50 μM. We also compared **L01** with other established OGT inhibitors such as BADGP and ST045849. At the same concentration (50 μM), **L01** showed stronger OGT inhibition than the other two compounds in Hela and K562 cells (Fig. S6). In immunocytochemistry analysis, **L01** treatment uniformly lowered O-GlcNAcylation within COS7 cells (Fig. 4b). This result was subsequently confirmed using click chemistry involving metabolic labeling of the O-GlcNAcylation protein with tetraacetylated N-azidoacetylglucosamine (Fig. 4c). We also observed that COS7 cells treated with 50 μM **L01** showed a time-dependent decrease in global O-GlcNAcylation (Fig. 4d). However, in a parallel test, OSMI-1 showed a more rapid onset of glycosylation reduction than **L01**. A significant reduction of O-GlcNAcylation was observed for **L01** only at 6 h, whereas OSMI-1 showed a similar effect within 2 h. In addition, **L01** also reduced global O-GlcNAcylation with similar effect as OSMI-1 at 50 μM in several other mammalian cell lines (293, Hela, K562, Fig. S6).

Having established that **L01** reduces global O-GlcNAcylation in cells, we next investigated O-GlcNAc levels on specific cellular markers after treatment with this compound by immunoprecipitating these proteins from cell lysates (Fig. 4e). As expected, Nup62 from untreated cells showed O-GlcNAc modification by using CTD110.6, whereas little O-GlcNAc modification of Nup62 was found in the cells treated with **L01**. Immunoprecipitated Nup62 was also probed with an O-GlcNAc Enzymatic Labeling System, and similar results were obtained by detection with streptavidin. Furthermore, **L01** caused Nup62 to shift to a lower molecular weight, consistent with a loss of the O-GlcNAc residues. OGA is also O-GlcNAcylated and OGA levels of are regulated by this modification. In this study, **L01** reduced cellular OGA levels without affecting cellular OGT levels. From Fig. 4e, we can also observe that **L01** was as effective as OSMI-1 in suppressing O-GlcNAcylation of specific targets in living cells. Therefore, these results demonstrate that **L01** inhibits OGT activity in cells.

Concerning the target promiscuity, lectins were used to analyze cell glycans following the treatment of cells with **L01**. Ten biotinylated lectins, Concanavalin A (ConA), Dolichos Biflorus Agglutinin (DBA), Ulex Europaeus Agglutinin (UEA), Jacalin (JAC), Soybean Agglutinin (SBA), Vicia Villosa Lectin (VVL), Lotus Lectin (LL), Peanut Agglutinin (PNA), Ricinus Communis Agglutinin (RCA) and succinylated Wheat Germ Agglutinin (sWGA), which recognize different features of N- and O-glycans, were employed to probe the glycan composition. COS7 cells were treated with 50 μM **L01** or OSMI-1 for 24 h. For a positive control, the levels of succinylated WGA (sWGA), which recognizes N-acetylglucosamine, were reduced to similar levels by either **L01** or OSMI-1 (Fig. 4f). No visible changes in bands detected by ConA, DBA (Fig. 4e), UEA, VVL, LL, PNA, RCA or SBA (Fig. S7) were observed in COS7 cells treated with both compounds. However, for JAC, **L01** but not OSMI-1 treatment caused slight changes in the glycans from COS7 cells (Fig. 4f), indicating that **L01** may block mucin-type O-glycan synthesis. Nevertheless, we confirmed that **L01** is an efficient, specific OGT inhibitor in cells.

### **L01** is a low toxicity OGT inhibitor

**Figure 5:**
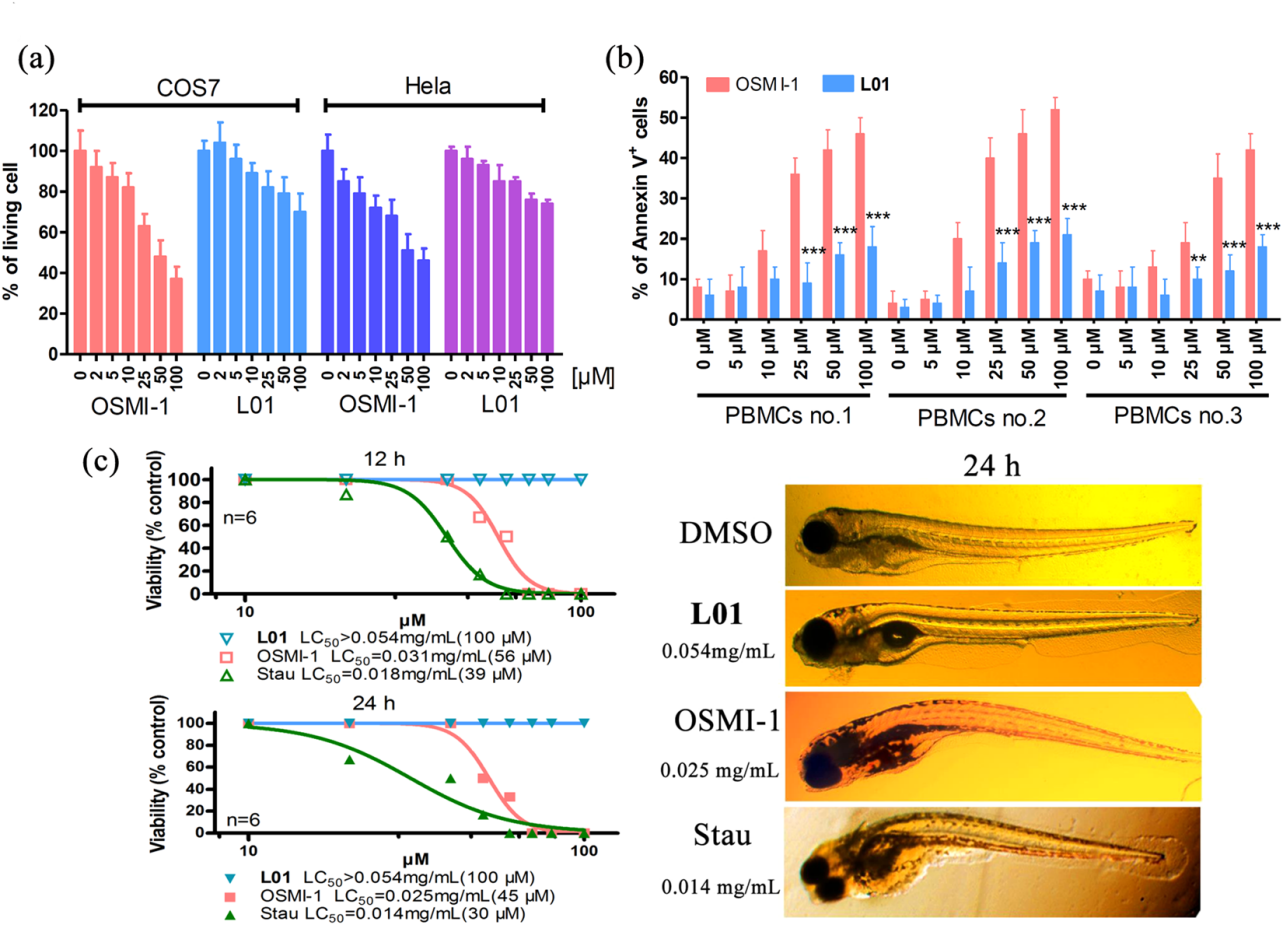
*In vitro* and *in vivo* toxicity of **L01**. (**a**) Effects of **L01** and OSMI-1 on cell viability. COS7 and Hela cells were treated with indicated doses of **L01** or OSMI-1 for 24 h. Cell viability was detected by CCK8 analysis (data represent mean ± s.e.m., n = 3). (**b**) Cytotoxicity of **L01** and OSMI-1 on PBMCs. Three primary cells from healthy donors were treated with increasing concentration of **L01** or OSMI-1, and Annexin V was measured by flow cytometry after 24 h. Data are the mean ± s.e.m. (n = 3). (**c**) LC_50_ of **L01** compared with OSMI-1 in zebrefish model. Morphogenesis of zebrafish embryos treated with **L01** at 0.054 mg/mL (100 μM) and OSMI-1 at 0.028 mg/mL (50 μM) for 24 h.

Most reported OGT inhibitors possess off-target toxicity^8^. To determine whether there were non-specific effects of **L01**, we first tested the effects of up to 100 μM **L01** on COS7 and Hela cells. Minimal dose-dependent effects on cell viability were observed after **L01** treatment for 24 h as shown by CCK8 analysis (Fig. 5a). We hypothesized that **L01** may reduce the cell proliferation by inhibiting O-GlcNAcylation of proliferation related proteins. By contrast, 50 μM OSMI-1 decreased the cell viability by more than 50%, indicating more side effects. To further evaluate **L01**’s specificity, three mononuclear cells isolated from primary normal peripheral blood mononuclear cells (PBMCs) were studied (Fig. 5b). Primary cells were treated with an increasing concentration of **L01** or OMSI-1, and Annexin V was measured by flow cytometry after 24 h. **L01** was relatively nontoxic towards normal PBMCs, whereas OSMI-1 showed a dose-dependent induction of cell death.

As a convenient and predictive animal model, the zebrafish model organism is increasingly used for assessing drug toxicity. Numerous studies have confirmed that mammalian and zebrafish toxicity profiles are strikingly similar, and zebrafish can usually serve as an intermediate step between cell-based evaluation and conventional animal testing^23,24^. In this study, the zebrafish model was used to investigate **L01** acute toxicity *in vivo* (Fig. 5c). The well-known toxic compound staurosporine (Stau) was used as a benchmark. Concentrations of **L01** from 0.0054 mg/mL (10 μM) to 0.054 mg/mL (100 μM) were investigated, and no zebrafish death was observed even at the highest dose at 12 and 24 h, while the LC_50_ of OSM1-1 was 0.031 mg/mL (56 μM, 12 h) and 0.025 mg/mL (45 μM, 24 h). From these data, we demonstrated that **L01** has low toxicity *in vitro* and *in vivo*.

## Discussion

Natural products offer a rich source of bioactive structural motifs for identifying novel low molecular weight lead structures that are active against a wide range of assay targets^15,17^. However, there have been no reports of natural product-like OGT inhibitors. In this study, we described a cell-permeable and relatively low toxicity small molecule OGT inhibitor, **L01**, which was identified through structure-based virtual screening from natural products. **L01** is an amentoflavone derivative and was reported as the major anti-inflammatory compound in the seed of *Semecarpus anacardium* ^25^. Amentoflavone also possesses several bioactivities, such as antitumor and antifungal effects^26,27^. Nevertheless, the mechanism by which amentoflavone exhibits its antitumor activity is not completely clear^28^. To the best of our knowledge, this class of compounds has not been reported as OGT inhibitors in the literature. O-GlcNAcylation is one of the most common posttranslational modifications and modulates the function of a wide-range of proteins, regulating various biological processes, such as gene expression, metabolism, signal transduction, cellular stress response and protein stability^2,3^. Given the complexity of OGT’s biochemical activities, inhibition of OGT by **L01** would interfere with other cellular processes beyond O-GlcNAcylation. This may partially explain the anticancer mechanism of **L01**’s analogue amentoflavone.

Even with its current limitations, in silico virtual screening offers a practical pipeline to discover new potential drug-like compounds for pharmaceutical research. Many novel scaffolds have been successfully discovered using structure-based computational analysis^29^. Based on high-resolution X-ray 3D crystal structures of OGT, structure-based virtual screening was employed. The workflow of our screening centered on chemical structure standardization and preparation, followed by careful evaluation of the sugar donor binding site of OGT in the receptor library. The multiple protein structures helped to realize the receptor flexible partially due to relatively independent and flexible side chains. We narrowed our interests from a total of 61 000 compounds to 200 top candidate compounds. Among these, 12 were tested for inhibition of OGT activity in HPLC based preliminary experiments. Of the candidate compounds tested, **L01** showed remarkable inhibition of OGT activity.

Based on the docking and molecular dynamics analysis, we anticipate that **L01** may form hydrogens bonds with several important residues in the UDP binding cavity of OGT to insert deeply into the cleft. Among these residues, Asn557 forms an important interaction with **L01**, which was not found in UDP (Fig. S5a). In virtual mutation simulation, **L01** tended to interact with Phe837 and Phe868 in OGT^N557A^ by hydrophobic and van der Waals interactions, but weakly interacted with the other part of the UDP binding cleft. However, this mutation did not significantly change the binding energy of UDP, suggesting the specificity of N557 in **L01** binding. As **L01** has larger topological polar surface area and higher rigidity than UDP, ring D of **L01** occupied the entrance of the UDP binding pocket between N557 and A897, which was difficult for UDP to reach. Further site-directed mutagenesis to target the potentially important OGT amino acid residue Asn557 followed by an *in vitro* glycosylation assay showed that N557A influences the binding of **L01** but not UDP. These data suggest that **L01** can bind to the UDP-GlcNAc-binding pocket, and Asn557 contributes to **L01**’s inhibitory activity. We hypothesize that N557 is a characteristic residue that differentiates the UDP binding pocket of OGT from others and might contribute to the specificity of **L01**. Thus, N557 might be a specific binding site for OGT inhibitor screening.

Multiple methods were used to verify the OGT inhibitory activity of **L01** *in vitro*. Similar IC_50_ values (21.8 μM and 20.2 μM) of **L01** were measured by UDP-Glo glycosyltransferase and HPLC assays, respectively. Although the binding sites of **L01** were similar to that of UDP, the binding free energy of **L01** was much higher than UDP. Thus, it is reasonable that the IC_50_ of **L01** is high. Notably, by using O-GlcNAc antibody CTD110.6, we demonstrated that this compound could inhibit O-GlcNAcylation of purified Nup62 by OGT in a cell-free system. To eliminate the nonspecific immunostaining of CTD110.6, O-GlcNAc Enzymatic Labeling System was employed. This methodology is highly specific, because the GalT (Y289L) in this system recognizes terminal GlcNAcs with high affinity^30^. Using this approach, inhibition of purified Nup62’s O-GlcNAcylation by **L01** was detected. IC_50_ values did not completely depend on UDP-GlcNAc concentration, indicating that **L01** did not fully act as a mimic of UDP-GlcNAc. This was consistent with its weak affinity to OGT. We noticed that the IC_50_ values increased in a stepwise fashion with the increasing UDP-GlcNAc concentration. **L01** was presumed to occupy the UDP-binding pocket of OGT and compete with UDP. However, our data suggested that **L01** does not completely act as predicted. We supposed that other binding sites near UDP might exist and influence the binding mode of **L01**. These assumed binding sites may affect the binding of UDP-GlcNAc directly. Further investigation of the binding mode and structure-activity relationship analyses are needed to understand its precise mechanism of OGT inhibitory activity. Additionally, on-target activity of **L01** was then detected in cells based on its ability to reduce cellular global O-GlcNAcylation; this compound inhibited O-GlcNAcylation of cellular Nup62, and reduced OGA protein levels. The reduction of global O-GlcNAcylation in several additional mammalian cell lines was also found by **L01** treatment.

Taken together, these data suggest that **L01** acts as an inhibitor of OGT activity by binding, at least in part, to the OGT substrate binding pocket. As described in the introduction, the potential non-specific effects of the developed OGT inhibitors caused their off-target toxicity. Target promiscuity of these inhibitors may contribute to their toxicity. Thus, targets of the same enzyme class are a particular concern in developing an on-target OGT inhibitor. ppGalNAcT2 is a member of the UDP-GalNAc polypeptide N-acetylgalactosaminyl-transferase (ppGalNAc-T) family of enzymes which transfers GalNAc to serine or threonine residues of proteins. This glycosyltransferase regulates the initial key steps of O-glycosylation^31^. **L01** could not inactivate ppGalNAcT2, suggesting **L01**’s selectivity and specificity for OGT. Probing with various lectins to detect the change of cell surface glycans following treatment of cells with the compound is another accepted strategy to evaluate the selectivity of glycosyltransferase inhibitors. Minimal interference with cell surface glycosylation was observed, indicating this compound did not substantially alter glycan composition.

To further assess **L01**’s on-target activity, the toxicity of this compound was tested *in vitro* and *in vivo*. First, in contrast to OSMI-1, **L01** had a slight effect on cell viability in a variety of mammalian cell lines, including tumor and normal cell lines. It was reported that 250 μM amentoflavone induced apoptosis in cancer cells^26^. We speculate that high concentrations of amentoflavone may enhance other side effects to induce cell death in cancer cells. Additionally, genetically ablating OGT in some cells led to their death^32^, suggesting that OGT plays a critical role in cellular physiology. However, the OGT functions that are essential for cell survival are not well understood. Based on different cell backgrounds, reduction of O-GlcNAc-modified proteins may lead to proliferation inhibition or modest cell death levels. Second, mononuclear cells isolated from primary normal PBMCs were used, and similar results were obtained. Finally, the zebrafish model was employed to investigate the acute toxicity of **L01**. In contrast to OSMI-1, **L01** presented low acute toxicity in the zebrafish model. From these data, we demonstrated that **L01** has on-target OGT inhibitory activity with low toxicity *in vitro* and *in vivo*.

In conclusion, we utilized structure-based virtual screening to discover a cell-permeable natural product inhibitor of OGT. **L01** specifically inhibited OGT activity in a cell-free system and reduced global O-GlcNAcylation in living cells, with potencies comparable to the well-known OGT inhibitor OSMI-1. Additionally, compared with other OGT inhibitors, **L01** showed low toxicity *in vitro* and *in vivo*, indicating its on-target inhibition of OGT. Our molecular modeling analysis suggests that **L01** might form multiple hydrogens bonds with OGT in the same positions as the sugar donor UDP. Notably, **L01** could bind to N557, which is near the UDP-binding pocket of OGT and might contribute to the specificity of **L01**. This study also validates the use of structure-based molecular docking to discover novel inhibitors of OGT. We envisage that **L01** may be employed as a useful scaffold for the development of more potent OGT inhibitors.

## Materials and Methods

### Cells and reagents

COS7, MCF10A, HeLa and K562 cells were obtained from Type Culture Collection of the Chinese Academy of Sciences (Shanghai, China). All cells were cultured in RPMI-1640 containing 10% fetal bovine serum at 37 °C with 5% CO_2_. Primary normal hematopoietic cells were obtained from three healthy volunteers donating their peripheral blood mononuclear cells (PBMCs). The authors confirm that all methods were carried out in accordance with written consent under approval of the Dalian University of Technology Review Board, that all experimental protocols were approved by the Medical Ethics Committee of School of Life Science & Medicine and that informed consent was obtained from all subjects. Mononuclear cells from peripheral blood samples were isolated by Ficoll-Hypaque sedimentation (Sigma Chemical Co., St. Louis, MO, USA). Cells were either used directly or cryopreserved in liquid nitrogen.

**L01** and other compounds were purchased from BioBioPha Co., Ltd. (Kunming, China). UDP-GlcNAc, OSMI-1 and BADGP were purchased from Sigma-Aldrich (MO, USA). ST045849 was purchased from TimTec (DE, USA). All compounds were solubilized in dimethyl sulfoxide (DMSO) to a storage concentration of 20 mM. The UDP-Glo assay kit was purchased from Promega (WI, USA). CKII peptide (KKKYPGGSTPVSSANMM) was synthesized by ChinaPeptides (Shanghai, China). Metabolic labeling reagents Click-iT GlcNAz, Biotin-alkyne, Protein reaction buffer kit and O-GlcNAc enzymatic labeling system were from Invitrogen (CA, USA). All the compounds were dissolved in DMSO.

### Molecular modeling

A total of 16 X-ray structures of human OGT (3PE3, 3PE4^19^, 3TAX^14^, 4AY6^33^, 4CDR^34^, 4GYW, 4GYY, 4GZ3^35^, 4N39, 4N3A, 4N3C^36^, 4XI9, 4XIF, 5BNW, 5C1D^37^, 5HGV^38^) bound to UDP, UDP-GlcNAc or an inhibitor from the Protein Data Bank were prepared as the receptor ensemble. The multiple receptor conformations for flexible docking is a practical shortcut to realize the receptor flexibility that could improve docking calculations^39^. These conformations exhibited considerable similarities, especially in the UDP binding pocket, but still had differences at the sidechains of many important residues (Fig. S5b). The 16 receptor structures were superimposed by UCSF Chimera to locate the same coordinate using a convenient docking box. A 22 × 22 × 22 Å box was set with the center next to the side chain of Pro559 to cover the UDP binding pocket of OGT.

Nearly 61,000 compounds from TCM Database@Taiwan were used as the ligand library. All molecules were predicted for ADME-Tox and other properties by the FAF-Drugs server. The whole library was filtered using the following properties: (1) 250 ≤ MW ≤ 600; (2) neutral and non-zwitterionic; (3) 2 ≤ ring systems ≤ 4; (4) hydrogen bond acceptors ≥ 1 and hydrogen bond donors ≥ 2. Concerning OGT conformational changes induced by UDP or other molecules, the free form structure of OGT was obtained, and no significant conformational change was found (Fig. S5c). Then, 4234 compounds were assessed by the virtual screening with the 16 conformations of OGT structures ensemble by Autodock Vina.

The average Vina scores of the lowest binding free energies for each ligand were re-ranked to obtain the compounds for further filter. The docking poses of Top 200 compounds with high average Vina scores were chosen for the ensemble cluster by UCSF Chimera. The biggest clusters of each compound were used for detailed research on the interaction between the ligand and receptors. The polar interaction with the key residues and regions, such as Q839, K842 and T921 in UDP binding pocket, were used as the necessary guideline. Meanwhile, the biological source and known bioactivity were used as reference.

All MD processes were performed by Gromacs 4.6.7 with an Amber99sb force field, and the parameters of ligands were calculated by Chimera and ACPYPE. The complex was solvated in a cubic TIP3P water box with 1 nm distance from the edge, which was neutralized by sodium ions. The temperature of the system was gradually heated to 300 K over 100 ps to perform the 5 ns NVT equilibration and 5 ns NPT equilibrations subsequently after two steps of energy minimization. Finally, the 10 ns MD simulations at 300 K and 1 atm were carried out with the LINCS algorithm to restrain the hydrogen positions at their equilibrium distances, which allowed the use of an integration time step of 2 fs. The binding free energy was calculated by the Molecular Mechanics-Poisson-Boltzmann Surface Area (MM-PBSA) method with g_mmpbsa, a plugin of Gromacs.

### Protein purification

The plasmids encoding OGT were a gift from Professor David J. Vocadlo (Simon Fraser University, Canada). Mutants (N557A and K898A) of OGT were generated using the QuikChange site-directed mutagenesis kit (Stratagene). The gene encoding Nup62 was subcloned into a pET28a vector. The expression and purification of Nup62, the full length OGT and its mutants were performed following previously described protocols^40^.

### Western/lectin blots

All biotinylated lectins were purchased from Vector Laboratories (CA, USA). The O-GlcNAc specific antibody CTD110.6 was purchased from Covance (WI, USA). Anti-OGT, anti-OGA, anti-Nup62 and anti-GAPDH antibodies were purchased from Cell Signaling Technology (MA, USA). HRP-conjugated goat anti-mouse IgM, goat anti-mouse IgG and goat anti-rabbit IgG were obtained from Santa Cruz Biotechnology (CA, USA). Streptavidin-HRP was purchased from Thermo (Shanghai, China). Samples were analyzed using standard procedures. Then, blots were developed with SuperSignal West Pico Chemiluminescent Substrate (Thermo).

### Immunocytochemistry

COS7 cells were fixed in 4% paraformaldehyde at 37 °C for 15 min and washed 3 times with PBS. Cells were permeabilized using 0.1% Triton X-100 in PBS-T for 0.5 h and blocked with 5% goat serum in 1% bovine serum for 1 h at room temperature. Then, COS7 cells were incubated with primary antibodies against O-GlcNAc (1:50, CTD110.6) diluted in 5% goat serum in 1% bovine serum overnight at 4 °C. Secondary antibody (goat anti-mouse IgM, conjugated to FITC) were used to visualize the proteins. Cells were cover slipped with Vectashield Mounting Medium with DAPI (Thermo) and mounted onto slides. Image acquisition was performed on a Leica confocal microscope.

### OGT activity assay

HPLC was used to preliminarily analyze the inhibition of OGT by the compounds. According to the previously described^41^, the reaction condition was optimized. 200 μM CKII, 300 nM OGT, 1 mM UDP-GlcNAc, the compounds (100 μM) and buffer (150 mM NaCl, 1 mM EDTA, 2.5 mM tris(hydroxypropyl)phosphine, 25 mM Tris-HCl, pH 7.4) were mixed and incubated at room temperature for 1 h. After being precipitated by methanol, the reaction mixtures were loaded onto HPLC (the reverse-phase chromatographic column was Zorbax SB-C18 StableBond analytical column, 250 mm × 4.6 mm, and a Zorbax SB-C18 analytical guard column, 4.6 mm × 12.5 mm, 5 μm, Agilent) to quantify the yield of glycopeptide product. Mobile phase A consisted of 0.1% TFA in H_2_O, and mobile phase B consisted of 0.1% TFA in MeCN. The components were eluted using a gradient (flow rate at 1 mL/min; at 0 min elution solvent mixture A/B = 90/10; at 20 min elution solvent mixture A/B = 70/30; wavelength = 214 nm). IC_50_ values were calculated using GraphPad 5 (n = 3).

A cell-free reaction system was used to determine the inhibition of O-GlcNAc level on a purified protein acceptor Nup62. Reaction mixtures containing 10 μM Nup62, 1 mM UDP-GlcNAc, 500 nM OGT, buffer (150 mM NaCl, 1 mM EDTA, 2.5 mM tris(hydroxypropyl)phosphine, 25 mM Tris-HCl, pH 7.4), and compounds were incubated at 37 °C for 1 h. Then, SDS-PAGE loading buffer was added and western blots were used to detect O-GlcNAc on Nup62.

OGT activity assay based on a UDP-Glo assay kit was performed as previously described^10^. Following the manufacturer’s protocol, assays were optimized and performed in white, flat bottom 384-well assay plate. CKII peptide was used as the acceptor. Reactions contained the following components: 250 nM OGT, 125 μM CKII and 40 μM UDP-GlcNAc, and buffer (150 mM NaCl, 1 mM EDTA, 2.5 mM tris(hydroxypropyl)phosphine, 25 mM Tris-HCl, pH 7.4). Luminescence was measured in triplicate using a microplate luminometer. IC_50_ values were calculated using GraphPad 5.

### Cell viability, cytotoxicity assay

Cell counting kit-8 (CCK-8) was used to evaluate the cell viability of cultured cells following the manufacturer’s protocol^42^. Inhibition rates were analyzed using GraphPad 5. PBMCs were treated with **L01** or OSMI-1 and cytotoxicity was assessed using Annexin-V in a FACSCalibur flow cytometry system.

### Acute toxicity assay

All procedures of the animal experiments were reviewed and approved by the Institutional Animal Care and Use Committee at the School of Life Science & Medicine, Dalian University of Technology and all experiments were conducted according to the relevant guidelines. Zebrafish embryos at 72-hours post-fertilization were selected for the acute toxicity assay. Zebrafish embryos were generated by natural pairwise mating and raised at 28.5 °C in embryo water. Zebrafish embryos were arrayed in 24-well plate (20 larvae per well) and incubated with 1 mL of embryo water per well containing various concentrations of **L01** or OSMI-1 at 28.5 °C for 24 h. DMSO (0.1%, v/v) solution served as the control. The observation of zebrafish was made directly in the 24-well plate using an inverted dissecting microscope. The number of dead zebrafish in each concentration solution was recorded within 24 h, and the survival rate was calculated (%).

### Statistical analysis

All data are presented as the mean ± s.e.m., n = 3. Data groups were compared by two-tailed Student’s t-test using the GraphPad Software. Differences between groups were considered statistically significant if *P* < 0.05. The statistical significance is denoted by asterisks (*P < 0.05; **P < 0.01; ***P < 0.001).

## Electronic supplementary material

Supporting Information
