# Supplementary material for: Discovery of a Low Toxicity O-GlcNAc Transferase (OGT) Inhibitor by Structure-based Virtual Screening of Natural Products
Source: Sci Rep. 2017 Sep 26;7:12334. doi: 10.1038/s41598-017-12522-0 (PMC5615061; doi:10.1038/s41598-017-12522-0)
Supplement: Supplementary file 1 — Supporting Information [file 41598_2017_12522_MOESM1_ESM.docx]

Discovery of a Low Toxicity O-GlcNAc Transferase (OGT) Inhibitor by Structure-based Virtual Screening of Natural Products

Yubo Liu^1^, Yang Ren^1^, Yu Cao^1^, Huang Huang^1^, Qiong Wu^1^, Wenli Li^1, 2^, Sijin Wu^2^, Jianing Zhang^1, *^

^1,^ School of Life Science & Medicine, Dalian University of Technology, Panjin, China.

^2,^ School of Life Science & Biotechnology, Dalian University of Technology, Dalian, China.

*Corresponding author: Jianing Zhang, School of Life Science & Medicine, Dalian University of Technology, Panjin, 122406, China; Tel.: +86 427 2631889; Fax: +86 427 2631889; E-mail address: [jnzhang@dlut.edu.cn](mailto:jnzhang@dlut.edu.cn)

Table S1 Structures of 12 which were selected out of 200 top-ranked compounds.

|  | ID | Vina Score (kCal/mol) | Structure |
| --- | --- | --- | --- |
| 1 | **L01** | -11.6 |  |
| 2 | **L36** | -11.2 |  |
| 3 | **L42** | -11.1 |  |
| 4 | **L73** | -10.9 |  |
| 5 | **L257** | -10.8 |  |
| 6 | **L299** | -10.19 |  |
| 7 | **L389** | -9.9 | 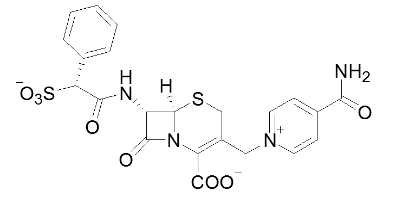 |
| 8 | **L566** | -9.8 |  |
| 9 | **L1945** | -9.79 |  |
| 10 | **L2899** | -9.61 |  |
| 11 | **L5733** | -9.45 |  |
| 12 | **L7721** | -9.42 |  |

Table S2 The Binding free energy results (MD) of OGT^WT/N557A^-**L01** and OGT^WT/N557A^-UDP complexes, which were decomposed to analysis the contribution of the energy terms.

| Contribution | WT-UDP | N557A-UDP | WT-**L01** | N557A-**L01** |
| --- | --- | --- | --- | --- |
| ∆E_vdW_ | -142.716 ± 1.419 | -121.224 ± 1.009 | -172.536 ± 1.053 | -214.084 ± 0.171 |
| ∆E_ele_ | -1011.324 ± 9.130 | -962.324 ± 6.272 | -126.719 ± 2.651 | -38.681 ± 2.095 |
| ∆G_PB_ | 614.861 ± 3.114 | 584.272 ± 5.122 | 218.403 ± 1.488 | 215.788 ± 2.452 |
| ∆G_SA_ | -15.665 ± 0.067 | -19.226 ±0.081 | -20.353 ± 0.067 | -22.865 ± 0.057 |
| ∆G_binding_ | -554.772 ± 6.934 | -518.632 ± 4.663 | -100.334 ± 1.352 | -60.005 ± 2.218 |

∆G_binding_ represents the binding free energy between ligand and protein. ∆E_vdW_, ∆E_ele_ represent the van der Waals and electrostatic contribution of molecular mechanics energy, respectively. ∆G_PB_, ∆G_SA_ represent polar and non-polar solvation contributions of the solvation free energy, respectively.

Fig. S1

(b)

(a)

(d)

(c)

Fig. S1 **L01** inhibited OGT in vitro. (a) HPLC-based assay was used to calculate the IC_50_ values of OSMI-1 and **L01** (0.2-167 μM, data represent the mean ± s.e.m., n = 3). (b) UDP-Glo assay was used to calculate the IC_50_ values of OSMI-1 and **L01** (0.25-500 μM, data represent the mean ± s.e.m., n = 3). (c) To prove **L01** is a reversible OGT inhibitor, the recoveries of the OGT catalytic activity after dilution of enzyme inhibitor complexes were examined. **L01** was combined and preincubated with OGT at concentrations of 10 × IC_50_ and 100 × IC_50_ for 0.5 h at room temperature (250 nM OGT, 150 mM NaCl, 1 mM EDTA, 2.5 mM tris(hydroxypropyl)phosphine, 25 mM Tris-HCl, pH 7.4). The reactions were subsequently diluted 100-fold to yield inhibitor concentrations of 0.1 × IC_50_ and 1 × IC_50_. 125 μM CKII and 40 μM UDP-GlcNAc was added. UDP-Glo assay was performed. For reversible enzyme inhibition, OGT activities were expected to recover to levels of approximately 90 and 50%, respectively, after dilution of the inhibitor to 0.1 × IC_50_ and 1 × IC_50_. OSMI-1 was used as a control (data represent the mean ± s.e.m., n = 3). (d) To prove **L01** is not an aggregator, small amounts of nonionic detergent and centrifugation were employed. **L01** was combined and preincubated with OGT at concentrations of 1 × IC_50_ and 10 × IC_50_ for 0.5 h at room temperature (250 nM OGT, 150 mM NaCl, 1 mM EDTA, 2.5 mM tris(hydroxypropyl)phosphine, 25 mM Tris-HCl, pH 7.4). Then 0.01% v/v freshly prepared Triton X-100 or 0.025% v/v Tween-80 was add to the reaction system. 10% m/v SDS was used as a negative control. Alternatively, the reaction system was centrifuged at 20,000 g for 10 min to get supernatant. 125 μM CKII and 40 μM UDP-GlcNAc were added. Nonionic detergent and centrifugation almost did not affect the inhibition activity of **L01**. By contrast, SDS reduced OGT activity significantly (data represent the mean ± s.e.m., n = 3). These data suggest that **L01** is not an aggregator.

Fig. S2

(a)

(b)

Fig. S2 (a) V_max_ for O-GlcNAcylation decreased with increasing **L01** concentration in the UDP-Glo assay. Reaction velocity plotted (the activity was expressed in relative light units (RLU)) against substrate concentration and fit to the Michaelis Menten equation. When using fixed saturating concentrations of CKII peptide, the V_max_ changed as a function of **L01** concentration (10 min incubation at room temperature, data represent the mean ± s.e.m., n = 3). The initial rate of the reaction was measured under low UDP-GlcNAc concentrations, the reaction rate increased as UDP-GlcNAc concentrations increase. However, as UDP-GlcNAc concentrations got higher, OGT became saturated with substrate and the rate reached V_max_. (b) Inhibition mode of wild type and N557A mutation OGT by **L01**. Various UDP-GlcNAc concentrations (2, 10, 25, 50, 100, 200 and 400 μM) were used in the UDP-Glo assay (data represent the mean ± s.e.m., n = 3).

Fig. S3

(a)

(b)

Fig. S3 (a) Inhibition activity of **L01** for ppGalNAcT2 at various concentrations. UDP-Glo based assay was used to calculate the IC_50_ values. Reaction conditions: 2 ng/μL ppGalNAcT2, 100 μM EA2 (peptide acceptor), 100μM UDP-GalNAc, 25 mM Tris-HCl (pH 7.4), 0.5 h incubation at 37℃ (data represent the mean ± s.e.m., n = 3). (b) To exclude the possibility that **L01** acts as an inhibitor through redox or other non-specific interaction, we tested the effect of **L01** on other cellular enzymes, such as glucose oxidase, lactic dehydrogenase and thioredoxin reductase. All these enzymes were recombinant expressed. The activity detection of these enzymes were performed as described previously ^[1-3](#_ENREF_1)^. No change of enzymatic activity was observed after **L01** treatment, suggesting that **L01** cannot interact with these proteins and affect their activity in a non-specific mode (data represent the mean ± s.e.m., n = 3).

Fig. S4


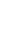

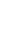

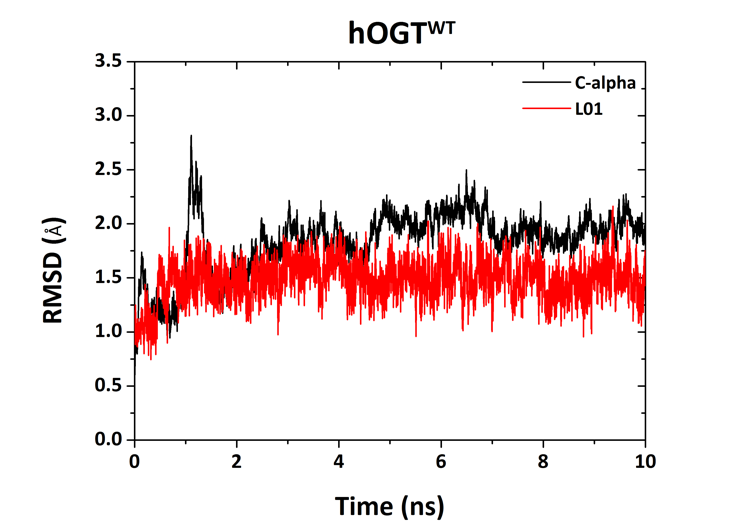

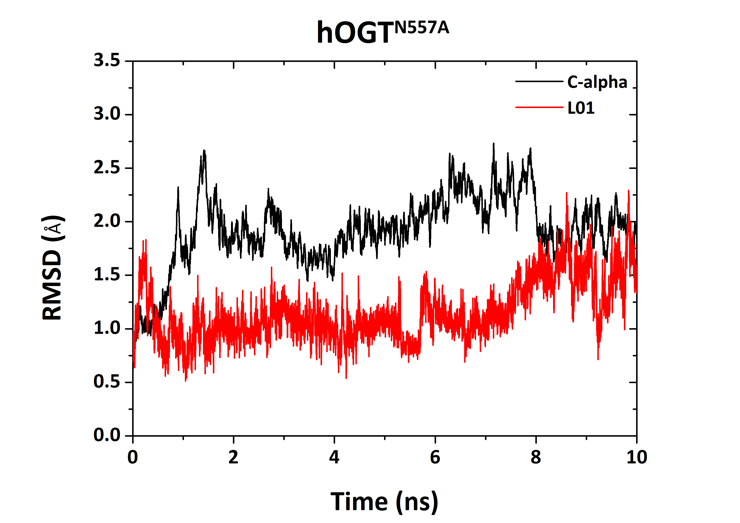


Fig. S4 The root-mean-square deviations (RMSD) of OGT^WT^ and OGT^N557A^ relative to their initial minimized complex structures as a function of time for OGT Cα atoms (black) and **L01** (red).

Fig. S5


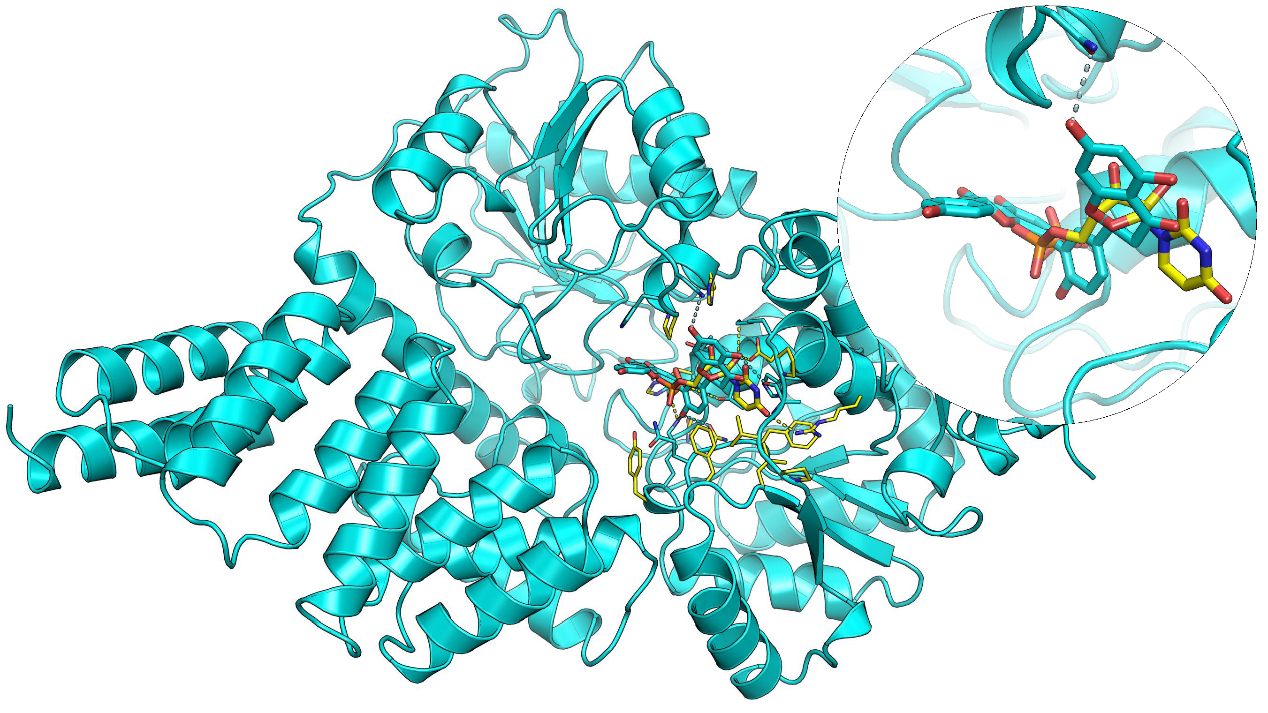


(a)

UDP

**L01**


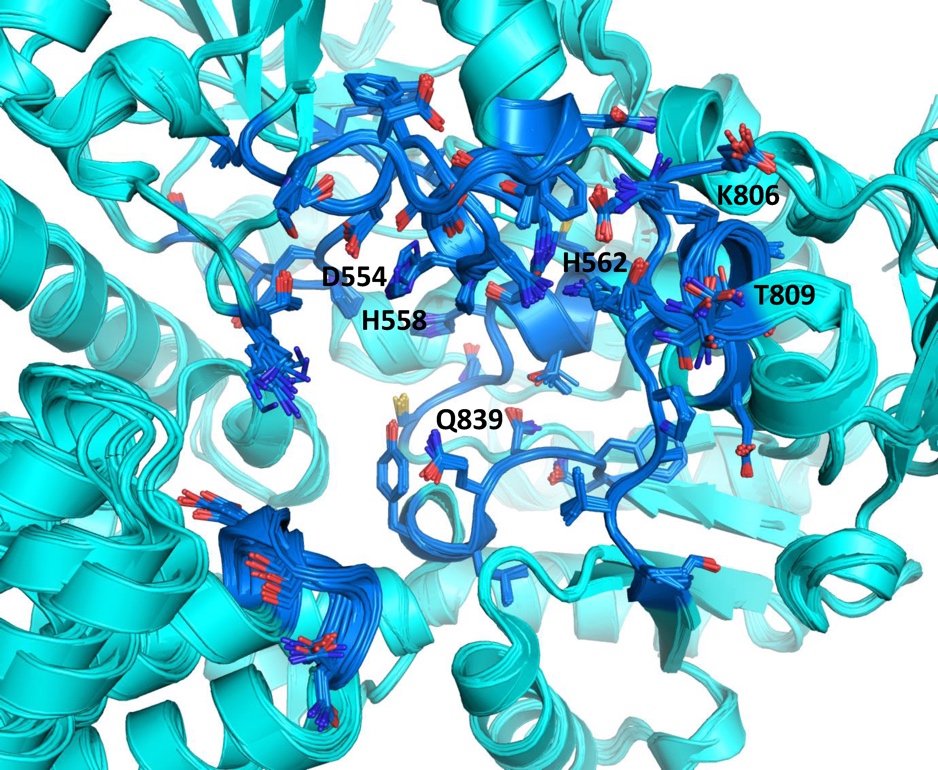


(b)


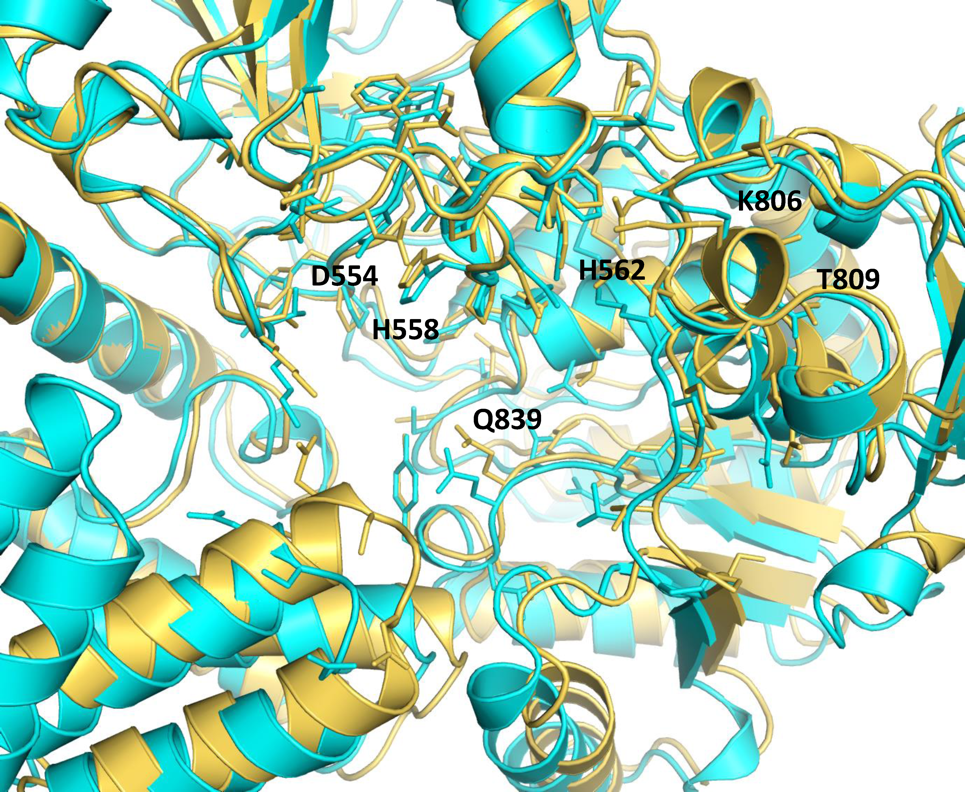


(c)

Fig. S5 (a) Molecular modeling of OGT (3PE3) with **L01** and UDP. (b) Conformational changes of the 16 receptors ensemble in local structure of UDP binding pocket. (c) Concerning OGT conformational change induced by UDP or other molecules, the free form structure of OGT was obtained. We calculated the free form structure of OGT by 100 ns molecular dynamics simulation from the crystal structure 3PE3. The final conformation of MD simulation showed that the main chain of the UDP binding pocket did not have significant conformational change. And the conformation changes of sidechain haven’t exceeded the range of the receptors ensemble which we found in local structure of UDP binding pocket (b

).

Fig. S6


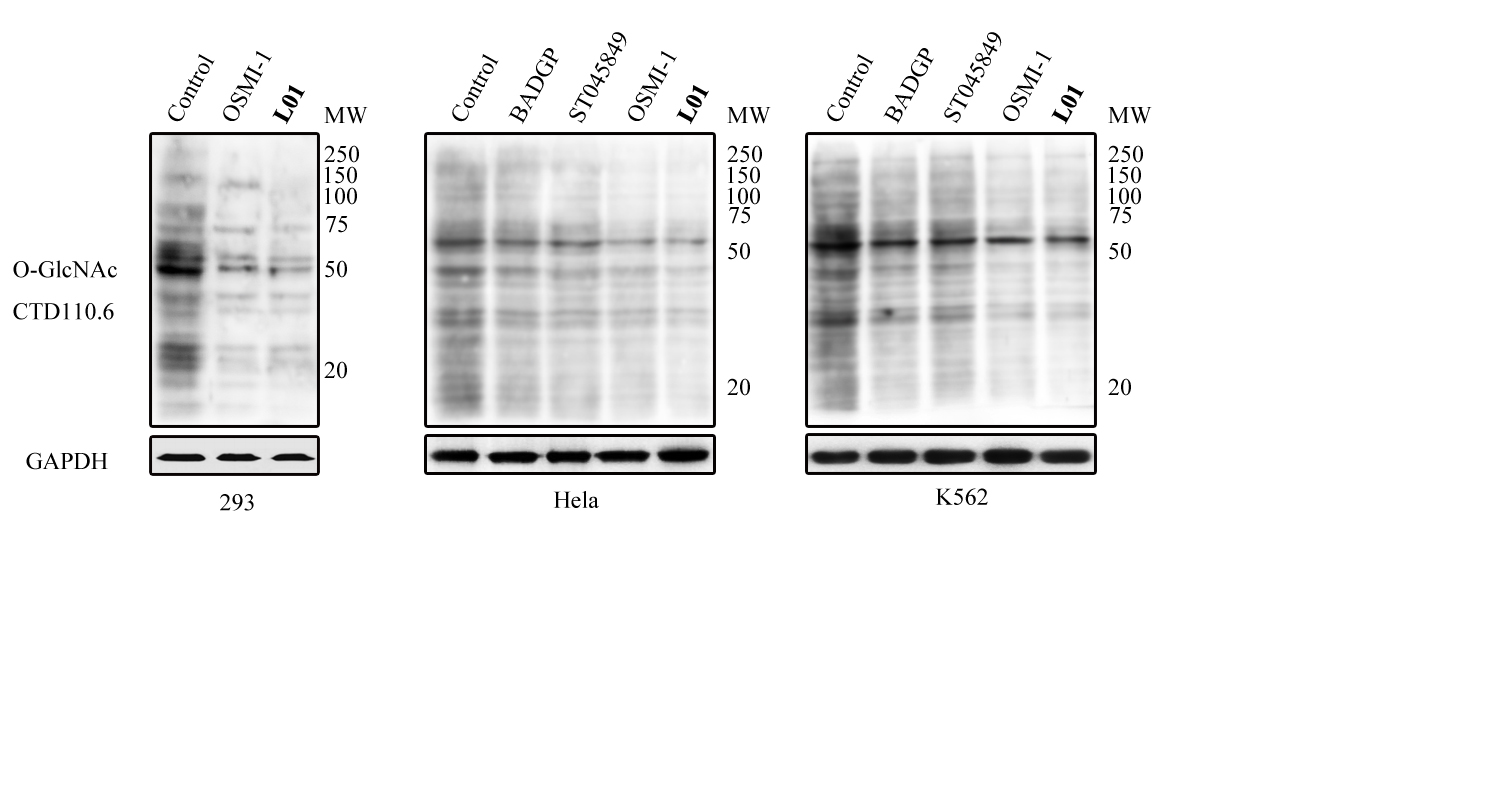


Fig. S6 Western blots of indicated cell lysates after different OGT inhibitors treatment at 50 μM for 24 h.

Fig. S7


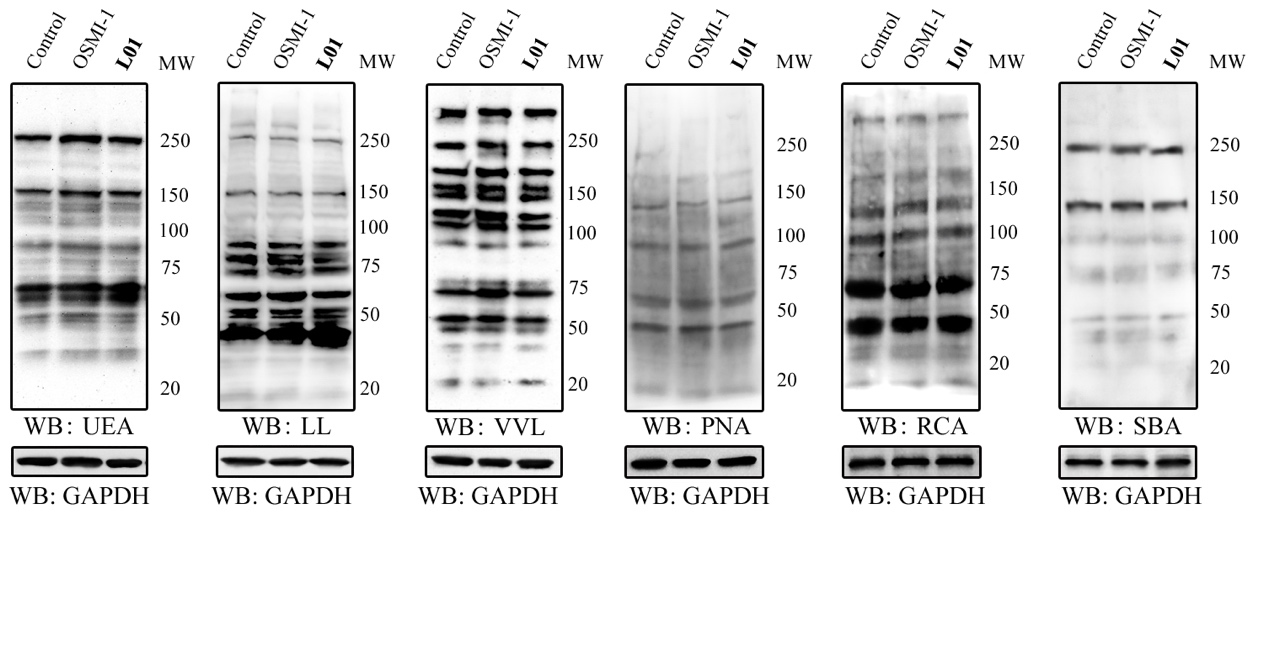


Fig. S7 Lectin blots of COS7 cell lysates after **L01** or OSMI-1 treatment at 50 μM for 24 h.

**References**

1 Lu, T., Peng, X., Yang, H. & Ji, L. The production of glucose oxidase using the waste myceliums of Aspergillus niger and the effects of metal ions on the activity of glucose oxidase. *Enzyme Microb. Technol.* **19**, 339-342 (1996).

2 Legrand, C. *et al.* Lactate dehydrogenase (LDH) activity of the number of dead cells in the medium of cultured eukaryotic cells as marker. *J. Biotechnol.* **25**, 231-243 (1992).

3 Tamura, T. & Stadtman, T. C. A new selenoprotein from human lung adenocarcinoma cells: purification, properties, and thioredoxin reductase activity. *Proceedings of the National Academy of Sciences* **93**, 1006-1011 (1996).
